# Drought and recovery effects on belowground respiration dynamics and the partitioning of recent carbon in managed and abandoned grassland

**DOI:** 10.1111/gcb.15131

**Published:** 2020-05-27

**Authors:** Johannes Ingrisch, Stefan Karlowsky, Roland Hasibeder, Gerd Gleixner, Michael Bahn

**Affiliations:** ^1^ Department of Ecology University of Innsbruck Innsbruck Austria; ^2^ Max Planck Institute for Biogeochemistry Jena Germany; ^3^ Leibniz‐Institute of Vegetable and Ornamental Crops Großbeeren Germany

**Keywords:** belowground carbon allocation, Birch effect, grassland abandonment, land‐use change, recovery, resilience, resistance, soil respiration

## Abstract

The supply of soil respiration with recent photoassimilates is an important and fast pathway for respiratory loss of carbon (C). To date it is unknown how drought and land‐use change interactively influence the dynamics of recent C in soil‐respired CO_2_. In an in situ common‐garden experiment, we exposed soil‐vegetation monoliths from a managed and a nearby abandoned mountain grassland to an experimental drought. Based on two ^13^CO_2_ pulse‐labelling campaigns, we traced recently assimilated C in soil respiration during drought, rewetting and early recovery. Independent of grassland management, drought reduced the absolute allocation of recent C to soil respiration. Rewetting triggered a respiration pulse, which was strongly fuelled by C assimilated during drought. In comparison to the managed grassland, the abandoned grassland partitioned more recent C to belowground respiration than to root C storage under ample water supply. Interestingly, this pattern was reversed under drought. We suggest that these different response patterns reflect strategies of the managed and the abandoned grassland to enhance their respective resilience to drought, by fostering their resistance and recovery respectively. We conclude that while severe drought can override the effects of abandonment of grassland management on the respiratory dynamics of recent C, abandonment alters strategies of belowground assimilate investment, with consequences for soil‐CO_2_ fluxes during drought and drought‐recovery.

## Graphical abstract

In a ^13^C‐tracer experiment we tested whether the response of carbon (C) dynamics to drought differs between managed and abandoned grasslands. Overall, drought reduced the amount of recent C respired belowground, and rewetting triggered a pulse of soil CO_2_ emissions, which was strongly fueled by recent C. Compared to abandoned grasslands, managed grasslands partitioned more assimilates to root storage and less to belowground respiration under ample water supply. Drought changed the patterns of belowground C partitioning, reflecting contrasting strategies of the two grasslands to enhance drought resistance and recovery.

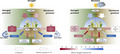

## 1 INTRODUCTION

Drought exerts strong effects on the terrestrial carbon (C) cycle (Frank et al., 2015; Reichstein et al., 2013; Sippel et al., 2018). It impairs the two largest fluxes of C between ecosystems and the atmosphere, i.e. photosynthesis and soil respiration. These fluxes are not independent, because rhizosphere respiration, i.e. respiration by roots and root‐associated microorganisms, relies closely on the supply of recent photoassimilates (Bahn et al., 2010; Trumbore, 2006). This link of assimilation and respiration represents a rapid pathway in short stature ecosystems, such as grasslands (Bahn, Schmitt, Siegwolf, Richter, & Brüggemann, 2009; Kuzyakov & Gavrichkova, 2010) where c. 12%–15% of assimilated C is respired belowground within days (Pausch & Kuzyakov, 2018).

Drought not only reduces the amounts of C taken up by plants, but can also alter belowground C allocation (Barthel et al., 2011; Burri, Sturm, Prechsl, Knohl, & Buchmann, 2014; Hagedorn et al., 2016; Hasibeder, Fuchslueger, Richter, & Bahn, 2015; Rühr et al., 2009), and can lead to a preferential allocation of newly assimilated C to root carbohydrate pools involved in osmotic adjustment and storage (Chaves, Maroco, & Pereira, 2003; Dietze et al., 2014; Hasibeder et al., 2015; Karlowsky, Augusti, Ingrisch, Hasibeder, et al., 2018; Volaire et al., 2020). While the concurrent effects of drought on the fate of recent C have been comparatively well studied, there is only very limited understanding of the postdrought dynamics of recent C during the recovery period. After rewetting, photosynthesis and soil respiration have been suggested to recover within days to weeks (Blessing, Barthel, Gentsch, & Buchmann, 2016; Ingrisch et al., 2018; Niboyet, Bardoux, Barot, & Bloor, 2017; Vicca et al., 2014; Zang et al., 2014). However, rewetting dynamics differ between these two processes, and dynamic shifts between C supply (photosynthesis) and demand (repair and regrowth; Karlowsky, Augusti, Ingrisch, Hasibeder, et al., 2018; Volaire et al., 2020; Zang et al., 2014) may change the fate of C from photosynthetic assimilation to soil respiration.

Soil respiration results from both root and microbial activity, the latter being supported by soil organic matter (SOM) and the supply of recent C from roots to the rhizosphere (e.g. Bahn et al., 2010). Rhizodeposition under drought is highly variable (Preece & Peñuelas, 2016), but in dry soils the plant–microbial C transfer is reduced (Fuchslueger, Bahn, Fritz, Hasibeder, & Richter, 2014; Karlowsky, Augusti, Ingrisch, Akanda, et al., 2018; Naylor & Coleman‐Derr, 2017) and recent plant‐derived C accumulates (Canarini, Kiær, & Dijkstra, 2017; Fuchslueger, Kastl, et al., 2014; Karlowsky, Augusti, Ingrisch, Akanda, et al., 2018; Manzoni, Schimel, & Porporato, 2012). Rewetting rapidly changes the physical conditions in the soil, thereby imposing stress on living organisms (Schimel, 2018) and increasing the accessibility and availability of C. This causes a transient pulse of soil respiration, the so‐called Birch effect, which can lead to distinct C losses from soil (Borken & Matzner, 2009; Kim, Vargas, Bond‐Lamberty, & Turetsky, 2012; Lopez‐Sangil, Hartley, Rovira, Casals, & Sayer, 2018; Vicca et al., 2014). To what degree such C losses affect the ecosystem C balance of the system in response to drought (Jarvis et al., 2007; Unger, Máguas, Pereira, David, & Werner, 2010), depends critically on the sources of C, i.e. whether it is derived from an active C fraction with rapid turnover or from old stable organic C pools. This is, however, still a subject of debate (Canarini et al., 2017; Lopez‐Sangil et al., 2018; Schimel, 2018), and highlights the need to understand the fate of recent C during rewetting.

European marginal grasslands, like mountain grasslands, shaped by socio‐ecological interactions and providing a variety of critical ecosystem services, are affected by recent and future socioeconomic as well as climatic changes (Egarter‐Vigl, Schirpke, Tasser, & Tappeiner, 2016; Lavorel et al., 2017; Schirpke et al., 2017). Reduced management intensity and abandonment, typical trends of land‐use change in marginal grasslands (Tasser, Leitinger, & Tappeiner, 2017), have been shown to alter grassland responses to drought (Stampfli, Bloor, Fischer, & Zeiter, 2018) by increasing the resistance and reducing recovery rates of productivity and respiration (Grime et al., 2000; Ingrisch et al., 2018; Lepš, Osbornová‐Kosinová, & Rejmánek, 1982). It has been suggested that such altered drought responses with abandonment could be due to shifts in plant‐community composition and plant–soil interactions. Abandonment favours slow‐growing plant species, which are often considered more stress‐tolerant, while fast‐growing species, typical of managed grasslands, are able to recover faster (deBoeck et al., 2018; Garnier et al., 2007; Mackie, Zeiter, Bloor, & Stampfli, 2018; Reich, 2014). Abandonment also favours fungal communities over bacterial ones; fungal communities and their networks are more stable under drought (de Vries et al., 2018), while bacterial communities turn over faster and release N upon rewetting, which can promote the recovery of fast‐growing plant species (Fuchslueger et al., 2019; Grigulis et al., 2013; Karlowsky, Augusti, Ingrisch, Hasibeder, et al., 2018). To date it is unclear how such changes in plant and microbial community structure triggered by abandonment affect belowground C allocation and the respiratory demand for recent assimilates. Even less is known on whether and how drought alters the effects of land‐use change on the utilization of recent C in soil respiration.

Here, we studied the dynamics of belowground respiration and the fate of recently assimilated C during and after a severe summer drought in a managed and an abandoned mountain grassland. We performed two ^13^CO_2_ pulse‐labelling campaigns during peak drought (i.e. the last week of the drought treatment) and the recovery phase and chased the tracer in soil respiration using isotope laser spectroscopy. We furthermore investigated how rewetting affected the fate of C, which had been photosynthetically taken up by plants and transferred to the soil during drought. We tested the hypotheses that: (a) drought reduces the temporal dynamics and the contribution of recently assimilated C in soil respiration during drought; (b) C assimilated during drought contributes significantly to C loss (Birch effect) upon rewetting; (c) due to the enhanced resistance of C dynamics in the abandoned grassland community, grassland abandonment reduces drought effects on C allocation to belowground respiration. Finally, we synthesized our findings on respiratory fluxes with previously published complementary data on C allocation to root carbohydrates obtained within the same experiment (Karlowsky, Augusti, Ingrisch, Hasibeder, et al., 2018) to obtain an integrated perspective and discussion on the effects of drought and abandonment on the belowground partitioning of recent C into respiration and storage.

## 2 MATERIAL AND METHODS

### 2.1 Study site

The study site is located near Neustift in the Austrian Central Alps (47°07′45″N, 11°18′20″E) and is part of LTER master site Stubai. It is composed of differently managed subalpine grasslands (Schmitt, Bahn, Wohlfahrt, Tappeiner, & Cernusca, 2010). Here, we study a managed hay meadow and an abandoned grassland, described in detail in Ingrisch et al. (2018). Briefly, the grasslands are situated on a southeast exposed hillside (c. 20°) with an average annual temperature of 3°C, an annual precipitation of 1,097 mm and soils are classified as dystric cambisol. The managed meadow (1,820 m a.s.l.) is lightly grazed in spring and autumn, mowed yearly in early August for hay harvest and periodically fertilized with manure. The vegetation is classified as *Trisetetum flavescentis*, consisting of perennial grasses and forbs. The abandoned grassland (1,970–2,000 m a.s.l., 47°07′31″N, 11°17′24″E) was abandoned in 1983. The vegetation is classified as *Seslerio‐Caricetum* with some dwarf shrubs (Grigulis et al., 2013; Schmitt et al., 2010). The abandoned grassland has generally a lower gross primary productivity (GPP) and aboveground net productivity (Ingrisch et al., 2018; Schmitt et al., 2010), holds a higher root biomass (Bahn, Knapp, Garajova, Pfahringer, & Cernusca, 2006; Karlowsky, Augusti, Ingrisch, Hasibeder, et al., 2018) and has higher SOM and lower nitrogen contents compared to the managed grassland (Fuchslueger, Bahn, et al., 2014; Meyer, Leifeld, Bahn, & Fuhrer, 2012).

### 2.2 Experimental set‐up and drought simulation

The study was conducted in a common garden at the managed grassland site. It is part of the larger experiment, the setting of which is described in detail in Ingrisch et al. (2018) and Karlowsky, Augusti, Ingrisch, Hasibeder, et al. (2018). Briefly, we extracted intact soil‐vegetation monoliths at both grassland sites 1 year before the experiment. The monoliths were fit into stainless steel cylinders (diameter 25 cm, height 28 cm), which had a reservoir for leachates at the bottom (Figure S2a, and detailed description in Obojes et al., 2015) and were buried in the soil at the managed grassland site. To avoid inflow of surface runoff, the cylinders were elevated 2 cm from the surrounding soil. The experiment was carried out in a full‐factorial design, crossing land‐use type (managed, abandoned) and treatment (control, drought) in a randomized block design (Figure S1). For this study, we used a subset of 24 monoliths to perform two pulse‐labelling campaigns (peak drought, recovery) with three replicates of each land use and treatment combination (2 campaigns × 3 reps × 2 land use × 2 treatment).

The drought simulation took place from 21 May 2014 to 28 June 2014. During this time, each block of monoliths was covered with rain‐out shelters. The tunnel‐shaped shelters had a base area of 3 × 3.5 m^2^ and a height of 2.5 m and were covered with light‐ and UV‐B permeable plastic foil (Lumisol clear AF; Folictec, light transmittance c. 90%). To enable air circulation inside the shelters, they were open at the bottom (up to 0.5 m) and at the top of the faces. Drought‐treated monoliths did not receive any water during the time of rain exclusion, while the control‐treated monoliths were watered manually with previously collected rainwater every 2–4 days. To avoid water limitation in the control treatment, soil moisture was monitored continuously (see Ingrisch et al., 2018) and the amounts of water added to controls were adjusted accordingly to maintain a soil moisture of c. 40 vol.‐%, the minimum during this period was 25 vol.‐%, which corresponds to a water‐filled pore space of c. 50%. During the first half of the rain exclusion, soil‐water content in the drought treatment declined to less than 20 vol.‐% in both grasslands and remained almost constant at this level in the following weeks (Ingrisch et al., 2018). To terminate the drought period (DOY 179), 50 mm of previously collected rainwater were added to each of the monoliths (drought and control treatments), to simulate a heavy rain event and achieve well‐defined rewetting. Irrigation water was always added slowly to the soil surface to ensure even percolation into the soil and to avoid runoff along the cylinder walls.

### 2.3 Pulse labelling

We performed two ^13^CO_2_ pulse‐labelling campaigns, the first during the last week of the drought treatment (‘peak drought’) and the second c. 2.5 weeks after end of the drought (‘recovery’). During each campaign, we labelled 12 monoliths, representing three replicates of each land use and treatment combination. Within each campaign, the labellings took place on 3 days, whereby on each day 4 monoliths representing each land‐use type and drought treatment were labelled (Figure S1). The ‘peak drought’ labelling took place on DOY 172–174 and the ‘recovery’ labelling and DOY 197, 199 and 200.

The labelling experiment and the procedure is described in detail in Karlowsky, Augusti, Ingrisch, Hasibeder, et al. (2018). Transparent acrylic glass chambers (diameter 25 cm, height 50 cm) were placed airtight on each monolith. The air inside the chambers was ventilated with fans and was temperature‐stabilized by pumping cold water through cooling tubes. Temperature inside the chambers was in the range of 25 ± 5°C. During labelling, we monitored air temperature, CO_2_ concentration and the ^13^CO_2_ isotope ratio (G2101i Analyzer; Picarro Inc.) and PAR (PQS 1; Kipp & Zonen). Pulse labelling was done on days with clear sky between 9:45 and 14:45 CET. Once the CO_2_ concentration in the closed chambers had dropped to c. 250 ppm, we added pulses of highly enriched ^13^CO_2_ (99 atom‐% ^13^C; CortecNet) with syringes, resulting in CO_2_ concentrations in the range of 400–800 ppm with approximately 50 atom‐% ^13^C. Each labelling lasted for 75 min.

### 2.4 Soil respiration and isotopic composition

We continuously measured soil respiration and its isotopic composition on the monoliths subject to pulse labelling in order to trace the belowground respiration of ^13^C tracer. Measurements on the ‘peak‐drought’ monoliths took place during the last week of the drought treatment until 3 days after the rewetting. Soil respiration chambers where then moved to the second set of monoliths (Figure S1), where they were employed from DOY 192 to 205.

We used a custom‐made automated set‐up that coupled 12 soil respiration chambers to an isotope analyser. Chambers were designed as steady‐state flow‐through chambers and were made from white PVC‐tubes with a diameter of 4.5 cm. They were open on the bottom, closed on the top and attached to a large inlet tube (diameter 3 cm) and an outlet tube (diameter 4 mm). The chambers were placed on bare soil at the centre of the monoliths, extending 2 cm into the soil (Figure S2a). The inlet was connected to a 50 L buffer volume to reduce fluctuations of CO_2_ concentrations in the air entering the chamber. Chambers were continuously flushed by drawing air via the outlet line at a constant flowrate of 170 ml/min to guarantee steady‐state conditions. The chamber design was tested for potential under‐pressure inside the chambers using a differential pressure transducer (MKS Baratron Type 226A Differential Pressure Transducer; MKS Instruments Ind.). Pressure effects were below the instrument resolution (<0.2 Pa) for flowrates up to 2 L/min.

All 12 chambers and their corresponding buffer volumes were connected to an automated multiplexer (Figure S2b), which switched one line towards the isotope analyser while flushing all other lines with the identical constant flowrate using an additional purge pump. Flowrate of the sample stream was logged every second with a mass flow meter.

The isotope analyser (G2101‐i Analyzer; Picarro Inc.) was operated in an air‐conditioned instrument shed next to the experimental plots. It continuously measured the concentrations of the isotopologues ^12^CO_2_ and ^13^CO_2_ with a precision of 200 and 10 ppb (30 s averaging), respectively, at a temporal resolution of c. 2 s (Picarro Inc., 2010). Each individual soil respiration measurement consisted of measuring isotopologue concentrations at the chamber outlet for 250 s, framed by measuring inlet air (from the buffer volume) for 100 s before and after. After each sequence of soil respiration measurements on all monoliths (c*.* 2 hr) three calibrations gases (400, 1,500 and 5,000 ppm CO_2_ in synthetic air) with known isotopic composition were measured. This allowed individual span‐offset calibrations for the isotopologues ^12^CO_2_ (range 400–5,000 ppm) and ^13^CO_2_ (range 4–50 ppm; Bowling, Sargent, Tanner, & Ehleringer, 2003).

### 2.5 Leachates

Leachates were sampled by completely emptying the water reservoirs of the monoliths before the drought treatment started, at peak drought (DOY 177), immediately after rewetting and 3 days after rewetting. Water volume was recorded and leachate samples were stored at −18°C prior to further analysis. For the analysis, ~ 1 ml of sample was filtered through prewashed (~0.5 ml of extract) 0.45 µm cellulose membrane filters (MULTOCLEAR 0.45 µm RC 13 mm; CS‐Chromatographie Service GmbH). To de‐gas the samples of inorganic C, filtered extracts were acidified with phosphoric acid to approx. pH 2 and gas‐flushed with N_2_ for 15 min. The degassed samples were then analysed as bulk fraction (no column) on high‐performance liquid chromatography (HPLC)—isotope ratio mass spectrometry (IRMS; Dionex UltiMate 3000 UHPLC coupled via a LC‐IsoLink system to a Delta V Advantage IRMS, Thermo Fisher Scientific). Each sample was measured in triplicate. Quality was controlled by repeated measurements of citric acid standards (δ^13^C = −18.58‰ vs. VPDB, Fluka Chemie AG; *SD* = 0.28‰, *n* = 48). Quantification was performed using a concentration row of the citric acid standard to calibrate the HPLC‐IRMS based on CO_2_ peak areas. Samples from unlabelled monoliths were used to obtain the natural abundance isotope composition of leached C.

### 2.6 Data analysis

Soil respiration rate (in µmol m^−2^ s^−1^) of each individual measurement was calculated as

(1) $$\text{SR}=\frac{f\cdot ({\text{CO}}_{2\text{out}}-{\text{CO}}_{2\text{in}})}{A},$$

where *f* is the flow rate through the chamber, CO_2out_ is the mean concentration measured at the chamber outlet, CO_2in_ is the mean concentration at the chamber inlet (in µmol/mol) and *A* is the area of the soil respiration chamber (m^2^).

The atom fraction of ^13^CO_2_ was calculated as:

(2) $$\chi {(}^{13}C)=\frac{{\;}^{13}{\text{CO}}_{2}}{{\;}^{13}{\text{CO}}_{2}{+}^{12}{\text{CO}}_{2}}.$$

The isotopic composition of soil respiration χ(^13^C)_SR_:

(3) $$\chi {{(}^{13}C)}_{\text{SR}}=\frac{\chi {{(}^{13}C)}_{\text{out}}\cdot {\text{CO}}_{2\text{out}}-\chi {{(}^{13}C)}_{\text{in}}\cdot {\text{CO}}_{2\text{in}}}{{\text{CO}}_{2\text{out}}-{\text{CO}}_{2\text{in}}},$$

where χ(^13^C)_in_ and χ(^13^C)_out_ denote the atom fraction of ^13^CO_2_ in the chamber inlet and outlet respectively. The fraction of ^13^C label in soil respiration was calculated as:

(4) $$\chi E{{(}^{13}C)}_{\text{SR}}=\chi {{(}^{13}C)}_{\text{SR}}-\chi {{(}^{13}C)}_{\text{SR(NA)}},$$

where χ(^13^C)_SR(NA)_ refers to the atom fraction of ^13^C in soil respiration before the labelling, corresponding to the natural abundance isotope composition of soil respiration.

The absolute rate of ^13^C label efflux in soil respiration (mg ^13^C m^−2^ hr^−1^) is calculated as:

(5) $$abs^{13}C=\chi E{{(}^{13}C)}_{\text{SR}}\cdot \text{SR}.$$

In order to estimate the error of soil CO_2_ efflux (SR), its isotopic composition (χ(^13^C)_SR_) and the amount of label recovered in soil respiration (*abs* ^13^C), the standard deviation of each of the measured variables in Equations (1)–(5) was propagated using first‐order Taylor expansion (Spiess, 2014; Ucar, Pebesma, & Azcorra, 2018). The coefficient of variation was calculated for SR and χ*E*(^13^C)_SR_ as ratio of propagated error and value. Measurements of SR and χ*E*(^13^C)_SR_ smaller than zero or with a coefficient of variation larger than 1 were excluded.

The fraction of incorporated tracer (*rel* ^13^C) in different compartments (carbohydrates, soil respiration) was calculated as the ratio of the absolute amount or rate of tracer efflux in this compartment (*abs* ^13^C) relative to total uptake of ^13^C label. The latter was calculated for each monolith as the sum of ^13^C incorporated in shoots and roots immediately after pulse labelling was ended (Karlowsky, Augusti, Ingrisch, Hasibeder, et al., 2018).

To enable grouping among replicates of the sequentially measured monoliths, we used spline functions to obtain timeseries of all monoliths with equal timestamps. Therefore, a spline function (R function ‘smooth.spline’, spar = 0.05) was fitted to each timeseries of CO_2_ and ^13^C‐tracer efflux rates. Based on these individual splines, efflux rates were predicted at a 2 hr interval. To avoid potential errors resulting from extrapolating splines over large data gaps and to keep this approach data‐driven, splines only filled data gaps with a maximum length of 6 hr.

We calculated the cumulative efflux of CO_2_ and ^13^C‐label for each monolith by integrating respiration rates and tracer efflux rates (*abs*.^13^C, *rel*.^13^C), respectively, following the trapezoid rule (Jurasinski, Koebsch, Guenther, & Beetz, 2014), i.e. by interpolating linearly between adjacent data points. For the cumulation, gaps within the time series were filled linearly, with exception of a single data gap of 24 hr within a subset of four monoliths that was filled with the mean cumulative efflux of the corresponding other replicates during this period. Cumulative effluxes were calculated for the 120 hr chase period after each pulse labelling and for the first 72 hr after end of the drought (rewetting). We estimated the accuracy of these cumulative fluxes for each chamber using a Monte‐Carlo analysis. Therefore, we generated 1,000 samples of each individual time series, by drawing samples for each individual measurement, based on its value and propagated error using the R function ‘rnorm’ and integrated each of the simulated time series as described above. In most cases, the variability between replicates was larger than the estimated uncertainty of individual monoliths, indicating that the biological variability exceeded the variability derived from methodological uncertainties.

To identify the potential effect of physical back‐diffusion of ^13^CO_2_ tracer on soil CO_2_ efflux dynamics after the pulse labelling (Burri, Sturm, Baur, et al., 2014; Subke et al., 2009), we performed a ‘dark pulse labelling’ on two additional grassland monoliths. It followed the same pulse‐labelling protocol, but the labelling chamber was darkened to exclude any photosynthetic uptake of ^13^C tracer. Immediately after the ‘dark labelling’, we placed soil respiration chambers on the monoliths and continuously measured soil‐CO_2_ and ^13^CO_2_ efflux, thereby quantifying the back‐diffusion of ^13^CO_2_ tracer from the soil. The efflux of tracer declined exponentially over time (Figure S3). We fitted an exponential model to this tracer efflux, which yielded a mean residence time of the tracer of 21–25 min (Table S1), which matches earlier estimates from the same grassland (Bahn et al., 2009). Thus, the effect of physical back‐diffusion on the shown soil ^13^CO_2_ dynamics was negligible.

The effects of drought, land use and their interaction on the cumulated respired CO_2_ and ^13^CO_2_ 120 hr after labelling were tested for each pulse‐labelling campaign separately using ANOVA (R base package, R Core Team, 2018) to report effect size *F*‐values and permutational ANOVA (package ‘lmPerm’; Wheeler & Torchiano, 2016) to obtain exact *p*‐values.

Effects of drought on the concentrations and amounts of organic C and ^13^C in leachates were tested with linear mixed‐effect models. We treated drought treatment, time (the leachate‐samplings right after rewetting and 3 days after rewetting), land use and the interactions of drought and land use and drought and time as fixed effects. Monolith identity was treated as random intercept to account for the replicated measurements on each monolith. All models were assessed for violations of homoscedasticity and normality. Models were fit using the lmer function from the package ‘lme4’ version 1.1‐18‐1 (Bates, Mächler, Bolker, & Walker, 2015). *p*‐values were obtained by Satterthwaite's method using the ANOVA‐function in the R package ‘lmerTest’ version 3.01 (Kuznetsova, Brockhoff, & Christensen, 2017).

## 3 RESULTS

### 3.1 ^13^CO_2_ emission dynamics during drought

**FIGURE 1:**
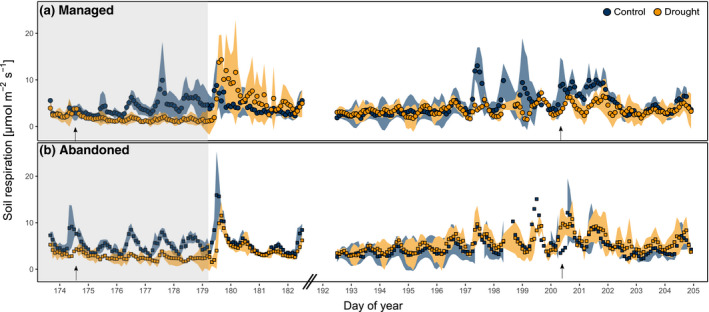
Time series of soil respiration in (a) the managed and (b) the abandoned grassland in the control (blue) and the drought (orange) treatment during the last week of the rain exclusion (grey shaded area) and the early recovery period. Arrows indicate the last days of each pulse‐labelling campaign. Blue and orange shaded areas indicate ±*SD*

**FIGURE 2:**
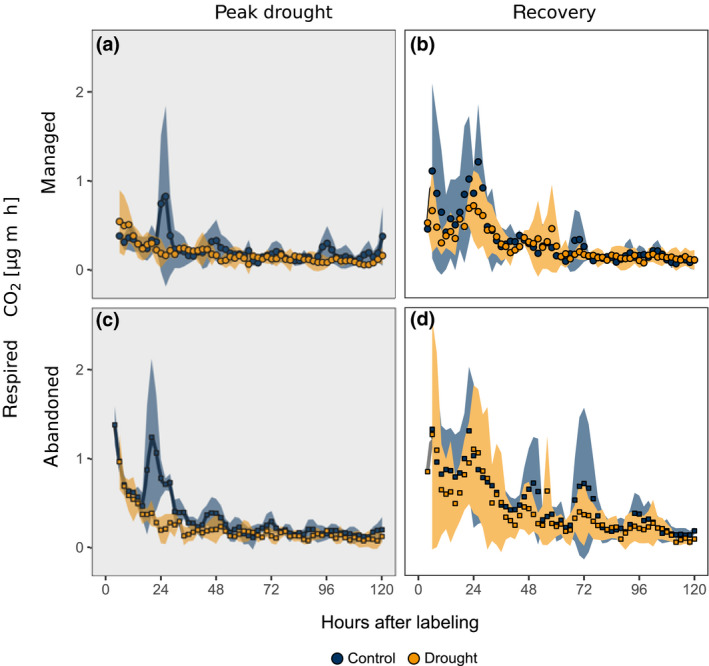
Time series of soil‐respired ^13^CO_2_ after pulse labelling during (a,c) peak drought and (b,d) recovery in the managed and abandoned grassland. Colours represent drought treatment. Blue and orange shaded areas indicate ±*SD*

**FIGURE 3:**
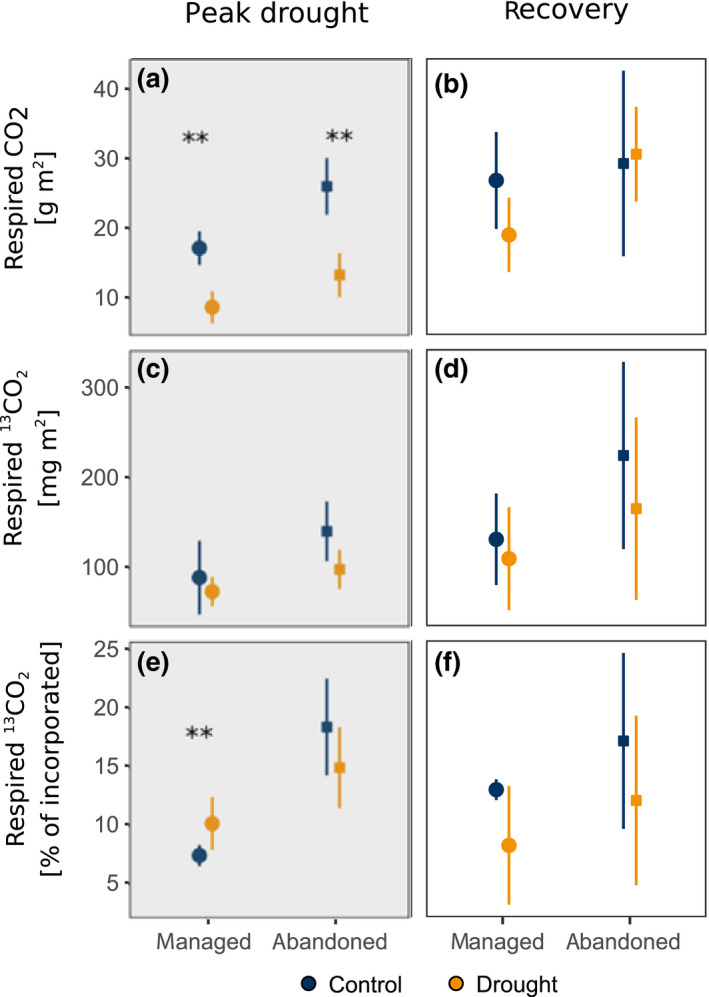
Soil‐respired CO_2_ and ^13^CO_2_ cumulated over the first 120 hr after the (a,c,e) peak drought and (b,d,f) recovery ^13^CO_2_‐labellings in both treatments. Points and error bars indicate mean ± *SD* across replicates. Asterisks indicate significant difference between drought and control treatment within land use (**p < .01, permutational one‐way ANOVA)

During drought, the uptake of C was reduced in both grasslands, reflected by a lower assimilation of ^13^C during labelling. This drought effect was more pronounced in the managed (−30%) compared to the abandoned grassland (−15%). Drought reduced soil respiration by 50% in both grasslands (Figures 1 and 3a,b; Table 1). Under both ambient and drought conditions ^13^C assimilated during labelling was rapidly (i.e. within 1.5 hr after start of the labelling) recovered in soil CO_2_ efflux and the rate of ^13^C efflux from soil declined in the following days (Figure 2). In the control treatments, the rate of ^13^C efflux showed diel patterns, with higher rates during noon, particularly on the first and second day after the labelling (Figure 2a,c). Under drought, the ^13^C efflux rate did not show any distinct diel fluctuations.

**TABLE 1:** Effects of drought, land use and the interaction on the cumulated amounts of respired CO_2_ and ^13^CO_2_, 120 hr after the peak drought and the recovery labelling, respectively, and in the first 70 hr after the rewetting

Campaign	Cumulation period		Amount			Respired ^13^C (absolute)			Respired ^13^C (relative)		
			*F* ^a^	*p*_F_ ^a^	*p*_exact_ ^b^	*F* ^a^	*p*_F_ ^a^	*p*_exact_ ^b^	*F* ^a^	*p*_F_ ^a^	*p*_exact_ ^b^
Peak drought + rewetting	Peak drought 120 hr	Drought	35.494	<.001	**<.001**	2.833	.131	**.040**	0.047	.834	.807
		Land use	14.362	.006	**.001**	4.930	.057	**.010**	21.286	.002	**<.001**
		Drought:Land use	1.412	.289	.240	0.501	.499	.335	3.306	.107	*.061*
	Rewetting 70 hr	Drought	0.881	.375	.242	0.599	.461	.307	7.161	.028	**<.001**
		Land use	0.069	.799	.774	0.571	.471	.320	1.842	.212	.110
		Drought:Land use	2.827	.131	**.002**	0.653	.442	.281	0.576	.470	.329
Recovery	Recovery 120 hr	Drought	0.419	.536	.382	0.722	.420	.233	2.148	.181	*.055*
		Land use	1.962	.199	*.063*	2.460	.155	**.046**	1.417	.268	.115
		Drought:Land use	0.836	.387	.226	0.157	.702	.586	0.002	.962	.946

^a^ *F*‐values and approximate *p*‐values from ordinary ANOVA.

^b^ Exact *p*‐values from the permutational ANOVA (function ‘aovp’, R package ‘lmPerm’), bold values *p*_exact_ < .05, italic values *p*_exact_ < .1.

Under ambient rainfall conditions, a significantly higher fraction of assimilated ^13^C was partitioned to belowground respiratory processes within the first 5 days after labelling in the abandoned (18.4 ± 4.15%) compared to the managed grassland (7.4 ± 0.85%, Figure 3e). Drought had contrasting effects on the fraction of respired ^13^C in the two grasslands: the fraction of tracer respired belowground increased in the managed (9.8 ± 2.2%), but not in the abandoned grassland (14.9 ± 3.4%; Figure 3e), which is also reflected in a weak statistical interaction of drought and land use (Table 1).

### 3.2 Effects of rewetting on fate of recent C

**FIGURE 4:**
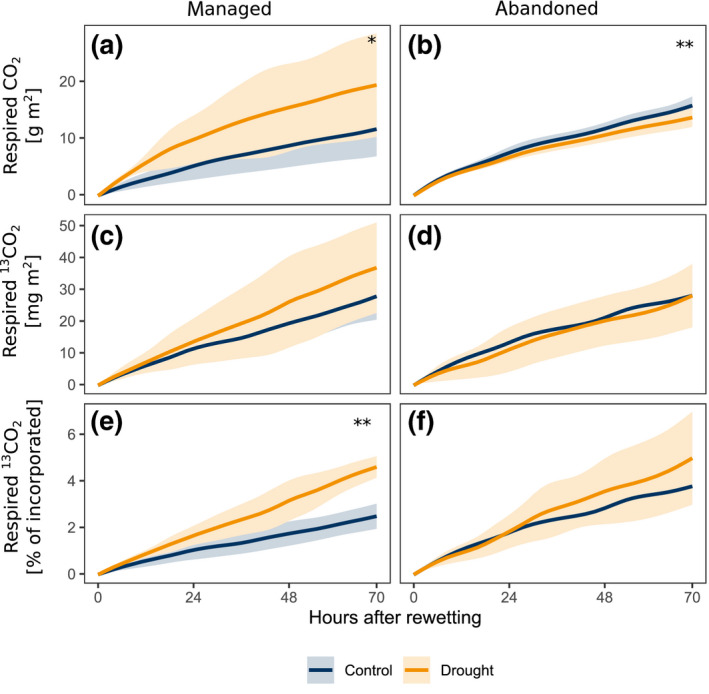
Cumulated efflux of soil‐respired CO_2_ and ^13^CO_2_ during the first 3 days after the rewetting in the (a,c,e) managed and (b,d,f) abandoned grasslands. Colours indicate drought treatment. Lines and shaded area indicate mean and *SD* across replicates. Asterisks indicate drought effect within land use (*p < .05, **p < .01, permutational one‐way ANOVA)

Upon rewetting of drought‐exposed monoliths, soil respiration rates increased rapidly, exceeding rates in control monoliths by up to a factor of three within 3 hr after rewetting and declining to control level within 2 days (Figure 1). Within 3 days after rewetting, a significantly larger fraction of ^13^C tracer assimilated during labelling was respired in drought‐exposed monoliths compared to controls (Figure 4e; Table 1).

**FIGURE 5:**
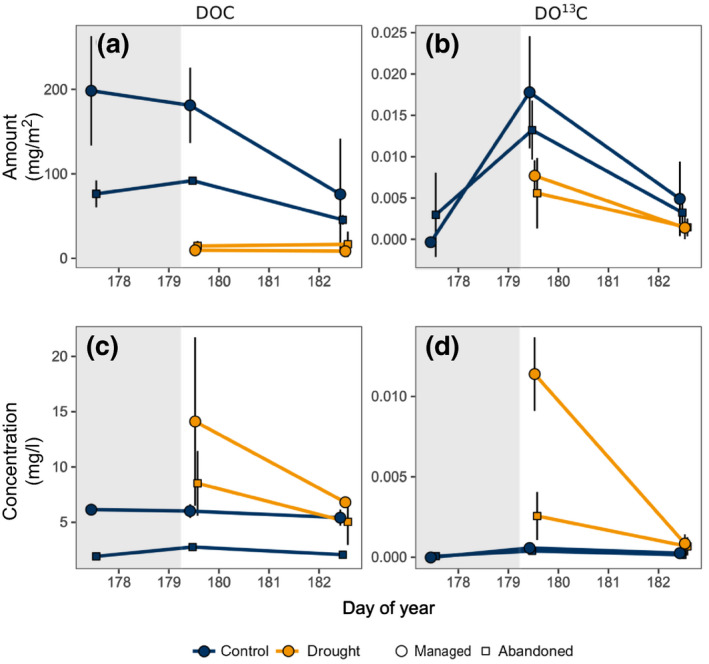
(a,b) Amounts and (c,d) concentrations of total dissolved organic C (DOC) and dissolved organic ^13^C (DO^13^C) in leachates in the managed grassland (circles) and the abandoned grassland (squares) at peak drought and after rewetting. Colours represent drought and control treatment. The shaded area indicates the peak‐drought period. Note that at peak drought there were no leachates. Error bars indicate ±*SD*

Drought‐treated monoliths leached less water, dissolved organic C (DOC) and labelled C (DO^13^C) in the days after rewetting than the controls (Figure 5). However, concentrations of DOC and DO^13^C recovered in leachates after the rewetting were significantly increased in the drought treatment, and quickly declined after rewetting (interaction of drought and time). Rewetting effects on soil respiration, DOC and DO^13^C in leachates were higher in the managed grassland than in the abandoned grassland (Figures 4 and 5; Table 2).

**TABLE 2:** Effects of drought, land use, sampling time (immediately after rewetting and 3 days after rewetting) and their interactions on the amounts and concentrations of C and ^13^C in leachates. Results from mixed‐model ANOVA. *F*‐values based on Satterthwaite's approximation, nominator and denominator degrees of freedom are given in subscripts. Superscripts represent respective *p*‐values. Bold values *p* < .05

Fixed effect	DOC amount	DO^13^C amount	DOC concentration	DO^13^C concentration
Treatment	***F*_1,8.2_ = 27.309^<.001^**	***F*_1,8.2_ = 9.856^.013^**	***F*_1,17_ = 19.868^<.001^**	***F*_1,17_ = 17.114^.001^**
Land use	*F*_1,8.2_ = 2.436^.156^	*F*_1,8.2_ = 1.023^.341^	***F*_1,17_ = 11.511^.003^**	***F*_1,17_ = 6.858^.018^**
Time	***F*_1,9.5_ = 21.140^.001^**	***F*_1,9.5_ = 60.114^<.001^**	***F*_1,17_ = 8.529^.010^**	***F*_1,17_ = 14.332^.001^**
Treatment:Land use	*F*_1,8.2_ = 4.125^.076^	*F*_1,8.2_ = 0.415^.537^	*F*_1,17_ = 0.007^.937^	***F*_1,17_ = 5.937^.026^**
Treatment:Time	***F*_1,9.5_ = 23.000^<.001^**	***F*_1,9.5_ = 9.680^.012^**	***F*_1,17_ = 5.171^.036^**	***F*_1,17_ = 11.746^.003^**

### 3.3 ^13^CO_2_ emission dynamics during recovery

During the recovery labelling, c*.* 2.5 weeks after end of the drought, plants of drought‐exposed monoliths assimilated a higher amount of ^13^C compared to controls (managed grassland +40%, abandoned grassland +5%). Diel dynamics in soil respiration quickly recovered after drought (Figure 1) and no drought effects on the amount of CO_2_‐efflux prevailed during the chase period of the recovery campaign (Figure 3b; Table 1). The dynamics of tracer efflux from soil were not affected by the previous drought treatment in both grasslands and showed diurnal patterns of ^13^C efflux in the days after the labelling (Figure 2b,d). There were no statistically significant effects of the previous drought treatment on the cumulated amount of respired ^13^C (Figure 3d,f; Table 1).

## 4 DISCUSSION

### 4.1 Metabolic utilization of recent assimilates during drought and recovery

**FIGURE 6:**
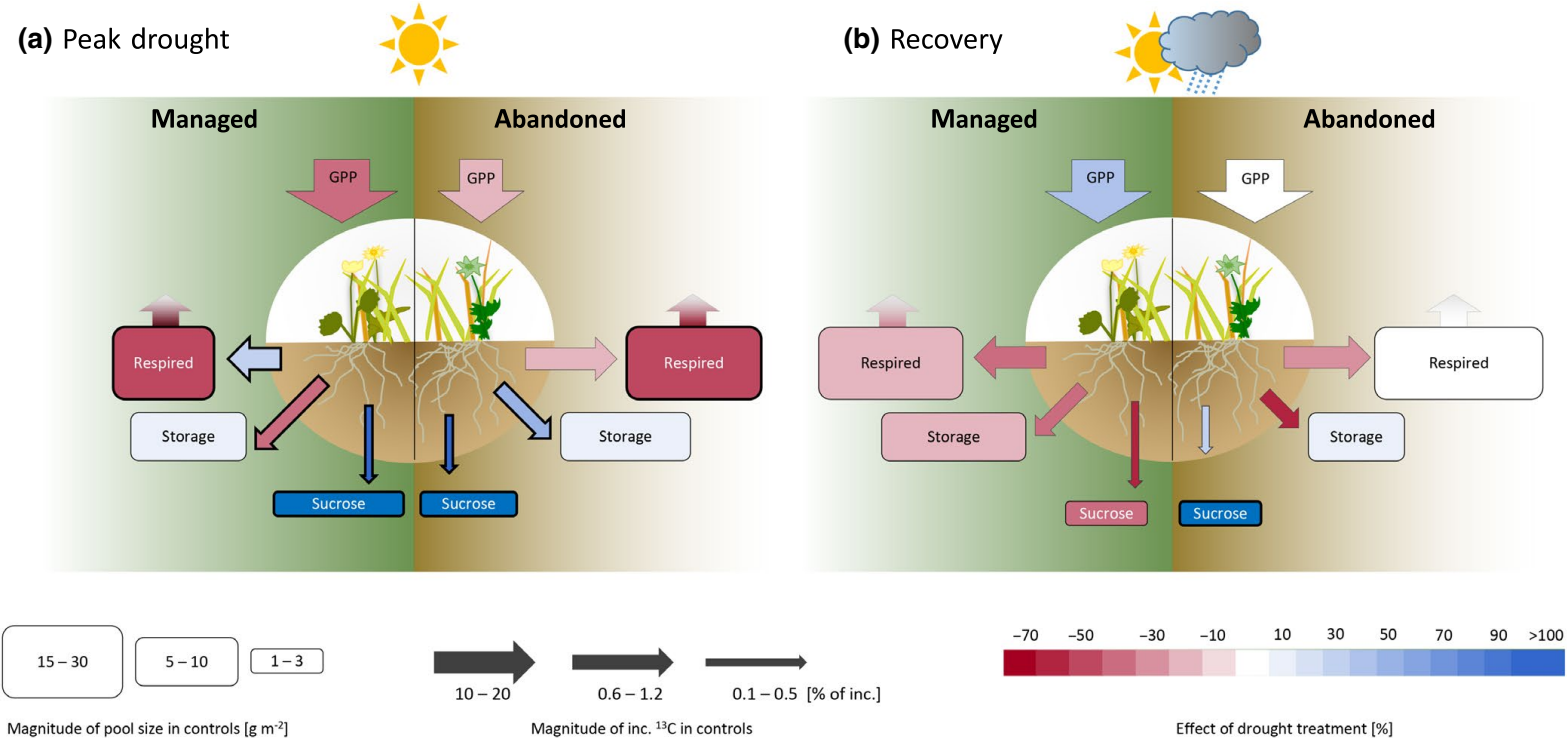
Synthesis of belowground carbon pools and fluxes and partitioning of ^13^C during the 120 hr chase period following the (a) peak drought and the (b) recovery labelling in the two grasslands based on the findings of this study and Karlowsky, Augusti, Ingrisch, Hasibeder, et al. (2018). Boxes refer to the amount of CO_2_ respired during the 120 hr and the carbohydrate content respectively. Arrows indicate the incorporation of ^13^C relative to initially assimilated ^13^C into each compartment. Sizes of the boxes and arrows indicate pool sizes and the amount of ^13^C recovered in the corresponding pools, respectively, under control conditions; the height of boxes and the width of arrows indicates size classes. Differences within each size class are indicated by box width and arrow length. Colours indicate direction and magnitude of the drought effects on each compartment and ^13^C allocation. Bold boxes and arrows indicate significant drought effects on the respective pool or flux within each grassland

Drought has been suggested to reduce GPP more strongly than soil respiration (Schwalm et al., 2010; Sippel et al., 2018), which raises the question whether and how drought affects the metabolic utilization of recently assimilated C belowground. In both the managed and the abandoned grassland, drought reduced C uptake (Figure 6) as well as soil respiration and the respiratory usage of recent C (Figure 3a,c) and dampened the diel dynamics in CO_2_‐efflux (Figure 1) and ^13^CO_2_‐efflux (Figure 2). Drought effects on belowground respiration can be attributed to reduced metabolic activity of both roots (Hasibeder et al., 2015; Lambers, Robinson, & Ribas‐Carbo, 2005; Sanaullah, Chabbi, Rumpel, & Kuzyakov, 2012) and microbes (Fuchslueger, Bahn, et al., 2014; Karlowsky, Augusti, Ingrisch, Hasibeder, et al., 2018). Drought effects on rhizosphere respiration can also be affected by the dynamics of assimilate supply to respiration (Barthel et al., 2011; Burri, Sturm, Baur, et al., 2014; Rühr et al., 2009), which was reflected by dampened diel cycles in ^13^CO_2_ efflux from soil (Figures 2 and 3). Similar patterns were reported from a grassland shading experiment, where interrupted C uptake altered the metabolic use of fresh and transitory carbohydrate pools, causing a cessation of diel fluctuations of respired tracer (Bahn et al., 2009). Similarly, it is likely that dampened tracer dynamics under drought were related to altered carbohydrate pool dynamics (Karlowsky, Augusti, Ingrisch, Hasibeder, et al., 2018). One indication of such drought‐induced changes in belowground carbohydrate dynamics is the preferential allocation of recent C to root sucrose (Hasibeder et al., 2015; Karlowsky, Augusti, Ingrisch, Hasibeder, et al., 2018, Figure S4; Figure 6), which is a primary precursor for root metabolism (Ghashghaie et al., 2003), but could also play an important role in osmoregulation during drought (Chaves et al., 2003; Hasibeder et al., 2015).

During recovery plants restored the assimilate supply to belowground respiratory utilization quickly. Two weeks after the drought had ended, the temporal dynamics as well as the cumulative amount of soil‐respired ^13^CO_2_ did not show any drought legacy (Figures 2 and 3). Interestingly, drought effects on the partitioning of assimilates into aboveground compartments or to the rhizosphere prevailed during this period of recovery (Karlowsky, Augusti, Ingrisch, Hasibeder, et al., 2018). This is in accordance with studies on short‐term postdrought recovery of young beech, reporting a rapid restoration (Blessing et al., 2016) and overcompensation of belowground C fluxes (Hagedorn et al., 2016), prior to a recovery of the C allocation into other plant compartments (Zang et al., 2014). Overall, these results demonstrate a high resilience of belowground metabolic functioning of grasslands even in the face of distinct direct effects of drought, driven through a rapid postdrought restoration of metabolic activity.

### 4.2 Rewetting triggers rapid metabolization of C assimilated during drought

Rewetting drastically alters the biophysical conditions in soils and forces plants and microorganisms to rapidly adjust C pools and cycling (Schimel, 2018), with distinct consequences for soil C losses (Borken & Matzner, 2009). Despite their importance for the overall C balance of drought events and the associated consequences for the global C cycle (Reichstein et al., 2013), the sources and drivers of these C losses are yet subject of debate (Canarini et al., 2017; Lopez‐Sangil et al., 2018). In our study we found that rewetting led to distinct respiratory losses of recent C from the ecosystem, since upon rewetting a significantly increased fraction of ^13^C taken up during peak drought was respired (Figure 4e,f; Table 1).

This recent C was derived from C pools that were built‐up during drought and became metabolically available for roots and microbes upon rewetting through different mechanisms. First, rewetting triggers a degradation of osmotic compounds in roots and microbes: Here, the high root sucrose concentrations maintained during drought for osmotic adjustment (see above) declined rapidly in the days following the rewetting (Figure S4; Table S3), presumably through metabolic utilization and/or root exudation. Similarly, as shown by earlier studies, microbes can rapidly adjust their osmotic potential upon rewetting (Borken & Matzner, 2009; Schimel, Balser, & Wallenstein, 2007; Warren, 2014). Second, soil microbes have been hypothesized to utilize rhizodeposits, which are either released by roots upon rewetting or which have accumulated during drought due to disrupted root‐microbial C transfer in dry soils (Canarini et al., 2017; Fuchslueger, Bahn, et al., 2014; Karlowsky, Augusti, Ingrisch, Akanda, et al., 2018). Here, the latter is evident from the increased concentrations of DOC and DO^13^C in soil leachates directly after rewetting (Figure 5), which demonstrates the existence of C pools in formerly dry soils that quickly get dissolved in soil solution. Overall, these results demonstrate that in addition to C derived from various SOM pools and plant and microbial litter (Borken & Matzner, 2009; Canarini et al., 2017; Lopez‐Sangil et al., 2018), in intact plant‐soil systems a significant fraction of the Birch effect is directly derived from plant‐C input during drought conditions and is thereby constituted of C with a short residence time in the system.

### 4.3 Abandonment alters assimilate partitioning under drought

The belowground allocation of recent assimilates differed between the two grasslands both under moist and drought conditions. Compared to the managed grassland, in the abandoned grassland a significantly larger fraction of recently assimilated C was respired belowground (Figure 3). This could be due to the larger root biomass and more pronounced plant–fungal interactions on the abandoned site (Bahn et al., 2006; Karlowsky, Augusti, Ingrisch, Hasibeder, et al., 2018) reflecting a response to reduced nutrient availability and a shift in plant functional composition (Grigulis et al., 2013; Zeller, Bahn, Aichner, & Tappeiner, 2000). In contrast, the managed grassland, composed of predominantly faster‐growing plant species (Grigulis et al., 2013; Ingrisch et al., 2018), holds larger root carbohydrate stocks and invests a larger fraction of assimilated C into root storage carbohydrates (Karlowsky, Augusti, Ingrisch, Hasibeder, et al., 2018), whereas belowground respiratory activity by roots and microbes is smaller.

These diverging belowground attributes of the two grasslands can also affect their drought resistance and recovery: A large belowground root and fungal network can improve water access and thereby support drought resistance of the abandoned grassland (de Vries et al., 2012; Karlowsky, Augusti, Ingrisch, Hasibeder, et al., 2018), whereas large root carbohydrate pools can potentially foster postdrought recovery (Hasibeder et al., 2015; Zwicke, Picon‐Cochard, Morvan‐Bertrand, Prud'homme, & Volaire, 2015). This is in line with the observed differences in resistance and recovery between the two grasslands.

In order to explore the overall effects of drought and grassland abandonment on the belowground partitioning of recently assimilated C between respiratory processes and carbohydrate storage, we integrated our findings with complementary data on ^13^C allocation to root carbohydrates obtained in the same pulse‐labelling experiment (Karlowsky, Augusti, Ingrisch, Hasibeder, et al. (2018); for methods and data expressed in units comparable to the current study see Supporting Information S1; Figure S4). We used the integrated dataset to test the hypothesis that under drought assimilates would be preferentially allocated to storage at the cost of metabolic utilization. Surprisingly, we found that under drought the grasslands showed diverging patterns of assimilate investment into belowground respiration versus storage. Under drought the managed meadow invested a larger portion of recent assimilates into belowground respiration, whereas the abandoned grassland invested less (Figure 6). Although we cannot distinguish between root and rhizomicrobial respiration here, the contrasting effects of drought on belowground metabolism are in line with other studies reporting variable effects of drought on rhizodeposition (Baptist et al., 2015; Preece & Peñuelas, 2016; Williams & de Vries, 2019). Noteworthy, these effects seem to be traded‐off against investment of resources into belowground storage, resulting in less allocation to storage in the managed and more allocation to storage in the abandoned grassland (Figure 6). Both of these contrasting assimilate‐investment strategies can contribute to enhancing the resilience (sensu Ingrisch & Bahn, 2018). To survive drought, plants need to balance resource allocation between reducing the immediate risk of mortality and maintaining or enhancing the ability for postdrought recovery. In this context, the per se more resistant community (abandoned grassland) increased its recovery capacity by investing into storage and the more rapidly recovering community (managed grassland) enhanced its resistance by investing into belowground metabolic activity. Therefore, these contrasting responses reflect strategies of optimal resource allocation to cope with limiting environmental conditions and are therefore in line with other trade‐offs reported from ecological systems, e.g. investments into growth versus defense (Herms & Mattson, 1992) or animal strategies for short‐ and long‐term survival in the presence of predators (McNamara & Buchanan, 2005).

While it can be assumed that the observed C allocation dynamics and their drought and recovery responses were shaped by the specific environmental conditions prevailing during the experiment, there is evidence that our findings can be generalized beyond our specific study. Previous experiments on the managed grassland under study indicated overall consistent dynamics of belowground C allocation across several years, both under control and under drought conditions (Bahn et al., 2009, 2013; Fuchslueger, Bahn, et al., 2014; Fuchslueger et al., 2016; Hasibeder et al., 2015). Also drought responses observed in other managed temperate grassland sites are consistent with our findings (Burri, Sturm, Prechsl, et al., 2014; Mackie et al., 2018) and suggest that our observations can be generalized beyond the weather‐ or site‐specific conditions. However, it should be acknowledged that drought timing, severity and post‐drought conditions are important and to date understudied constituents of drought‐ and drought recovery‐responses (Felton, Slette, Smith, & Knapp, 2020; Schwalm et al., 2017; Sippel et al., 2018; Song et al., 2019). Further studies are needed to elucidate their implications for carbon allocation and the carbon cycle in general.

The two grasslands studied here differ in their general strategies of growth and resource acquisition, which are characterized by a gradient of fast‐growing (managed grassland) versus slow‐growing plant communities, associated with bacterial‐ versus fungal‐dominated microbial communities (Grigulis et al., 2013; Ingrisch et al., 2018). Our study suggests that along with these changes in the fast–slow plant economic spectrum (Reich, 2014), abandonment also shifted the preferential allocation of assimilates from storage towards metabolic activity under ample water supply. Furthermore, these changes alter the importance of belowground metabolic activity under drought conditions, with consequences for short‐term losses of assimilated C during drought and rewetting. In the managed grassland dominated by fast‐growing species, increased investment of assimilated C into belowground metabolism during drought and rewetting increased the losses of recent C, whereas enhanced partitioning into storage in the abandoned grassland dominated by slow‐growing species supported a preservation of recent C. Overall, we conclude that while severe drought can override the effects of abandonment of grassland management on the respiratory dynamics of recent C, abandonment alters strategies of belowground assimilate investment, with consequences for soil‐CO_2_ fluxes during drought and drought recovery.

## CONFLICT OF INTEREST

The authors declare no competing interests.

## AUTHOR CONTRIBUTION

M.B. and G.G. conceived the study. J.I., S.K. and R.H. performed the experiment and collected data. J.I. and S.K. analysed the data. J.I. and M.B. led the writing of the manuscript. All authors contributed critically to the drafts and gave final approval for publication.

## Supporting information

Supplementary Material

## Data Availability

The processed data that support the findings of this study are openly available in the Zenodo repository under https://doi.org/10.5281/zenodo.3757939
