# Supplementary material for: Drought and recovery effects on belowground respiration dynamics and the partitioning of recent carbon in managed and abandoned grassland
Source: Glob Chang Biol. 2020 May 27;26(8):4366–78. doi: 10.1111/gcb.15131 (PMC7384171; doi:10.1111/gcb.15131)
Supplement: Supplementary file 1 — Supplementary Material [file GCB-26-4366-s001.docx]

**Drought and recovery effects on belowground respiration dynamics and the partitioning of recent carbon in managed and abandoned grassland**

## Supporting Information

Ingrisch Johannes, Karlowsky Stefan, Hasibeder Roland, Gleixner Gerd, Bahn Michael

**Table of Contents**

**S1:** Extended Methods: Plant carbohydrate data

**Fig. S1** Arrangement of grassland monoliths in the common garden.

**Fig. S2** Illustration of monolith containers and soil-respiration setup.

**Fig. S3** ^13^CO_2_ back-diffusion from soil after dark-labeling.

**Fig. S4** Root carbohydrates and incorporation of ^13^C.

**Table S1** Model parameters of ^13^CO_2_ back-diffusion.

**Table S2** Effect of drought, land use and interaction on carbohydrates and incorporated ^13^C.

**Table S3** Effects of rewetting on root carbohydrates and incorporated ^13^C**.**

## S1: Extended Methods: Plant Carbohydrate data

Within the same experiment, Karlowsky et al. (2018a) measured allocation of ^13^C to root carbohydrates. This was done on the identical set of monoliths and during the same pulse-labeling campaigns as described in this study. Their data is accessible via the DRYAD repository (Karlowsky et al., 2018b). We use data on the concentration of and the amount of incorporated ^13^C in root sucrose, root starch and root fructan for each monolith 120 hours after each labeling in order to obtain an overall synthesis on belowground plant partitioning of recent C. Next to the data described in Karlowsky et al. (2018a), the repository contains additional root carbohydrate data for the first set of monoliths (peak-drought labeling) measured 3 days after the rewetting, thereby allowing to assess C dynamics in these pools in response to rewetting.

Carbohydrate concentrations (mg g_dry mass_^-1^) were recalculated to obtain carbohydrate content per ground area (g m^-2^) using the corresponding root biomass (related to the upper 7 cm of soil, also available in Karlowsky et al. (2018b).

The effects of drought, land use and their interaction on carbohydrate contents and incorporated ^13^C (sucrose, starch + fFructan) 120 hours after labeling were tested for each pulse labeling campaign separately using ANOVA (R base package, R Core Team, 2018) to report effect size F-values and permutational ANOVA (package ‘lmPerm’ Wheeler & Torchiano, 2016) to obtain exact p-values.

Effects of rewetting on concentrations and ^13^C incorporation into root sucrose and root storage (starch + fructan) were tested using linear-mixed-effect models. We treated drought treatment, time (before and after rewetting), land use and the interactions of drought and land use, and drought and time as fixed effects. Monolith identity was treated as random intercept to account for the replicated measurements on each monolith. All models were assessed for violations of homoscedasticity and normality. Models were fit using the lmer function from the package ‘lme4’ version 1.1-18-1 (Bates, Mächler, Bolker, Walker, 2015). P-values were obtained by Satterthwaite’s method using the anova-function in the R-package ‘lmerTest’ version 3.01 (Kuznetsova, Brockhoff, Christensen, 2017).

**Fig S1: Arrangement of grassland monoliths in the common garden** at the managed grassland site. Shaded monoliths were used for the ^13^CO_2_ pulse-labeling in this study. Monoliths were arranged in randomized block design using six rain-out shelters (RS 1 to RS 6). Letters in monoliths denote days when labeling took place. Soil respiration instrumentation was operated in a climatized instrument shed.

**
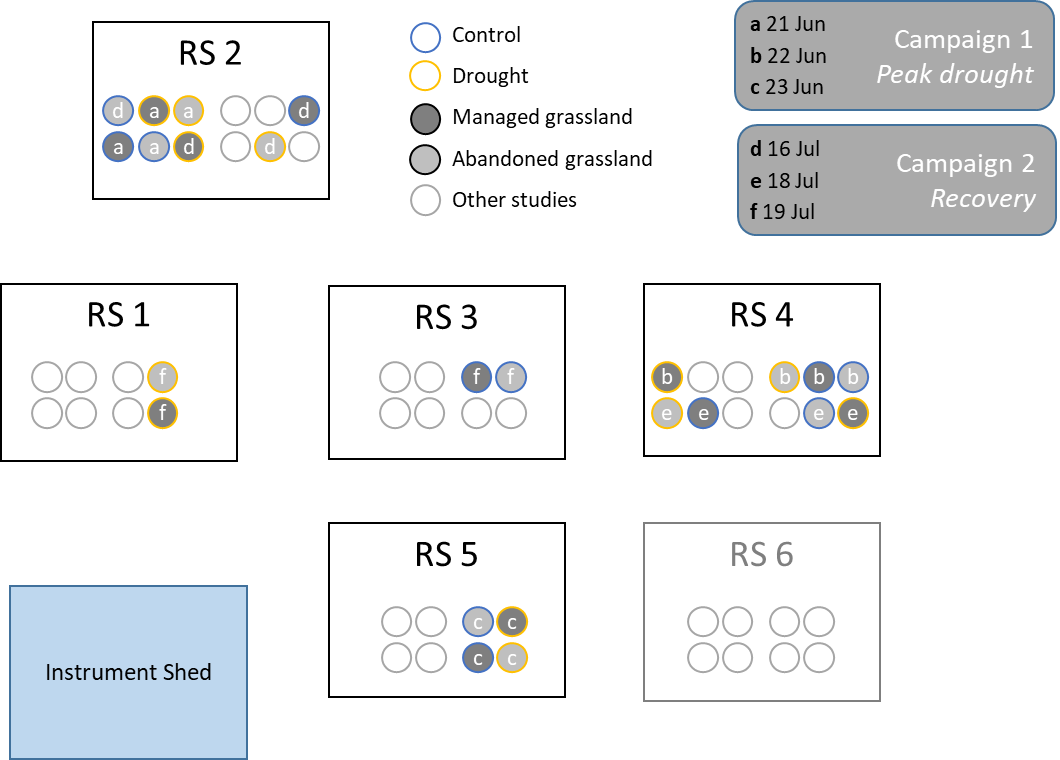
**

**Fig S2: Illustration of monolith containers and soil-respiration setup**. (A) Soil monoliths were placed in stainless steel cylinders and buried in the ground. A leachate reservoir at the bottom was accessible via a pipe. The upper edge of monolith containers was two centimeters above soil surface to avoid inflow of surface runoff. In each monolith, a soil-respiration chamber was placed in the center. The chamber inlet was a thick tube (diameter 3cm) that was connected to a buffer volume. Both, the chamber outlet as well as the buffer volume were connected to the multiplexer of the automated soil respiration setup. (B) Each of the 12 chamber inlet and outlet lines is connected to a custom-made multiplexer. This multiplexer directs one of the gas lines to the sample stream, while maintaining a constant flushing of all soil respiration chambers to maintain the chambers in steady-state conditions. The CO_2_-analyzer is located in an air-conditioned instrument shed. The analyzer is calibrated regularly with three calibration gases. The flowrate of the sample stream is logged with a mass-flow controller (MFC).


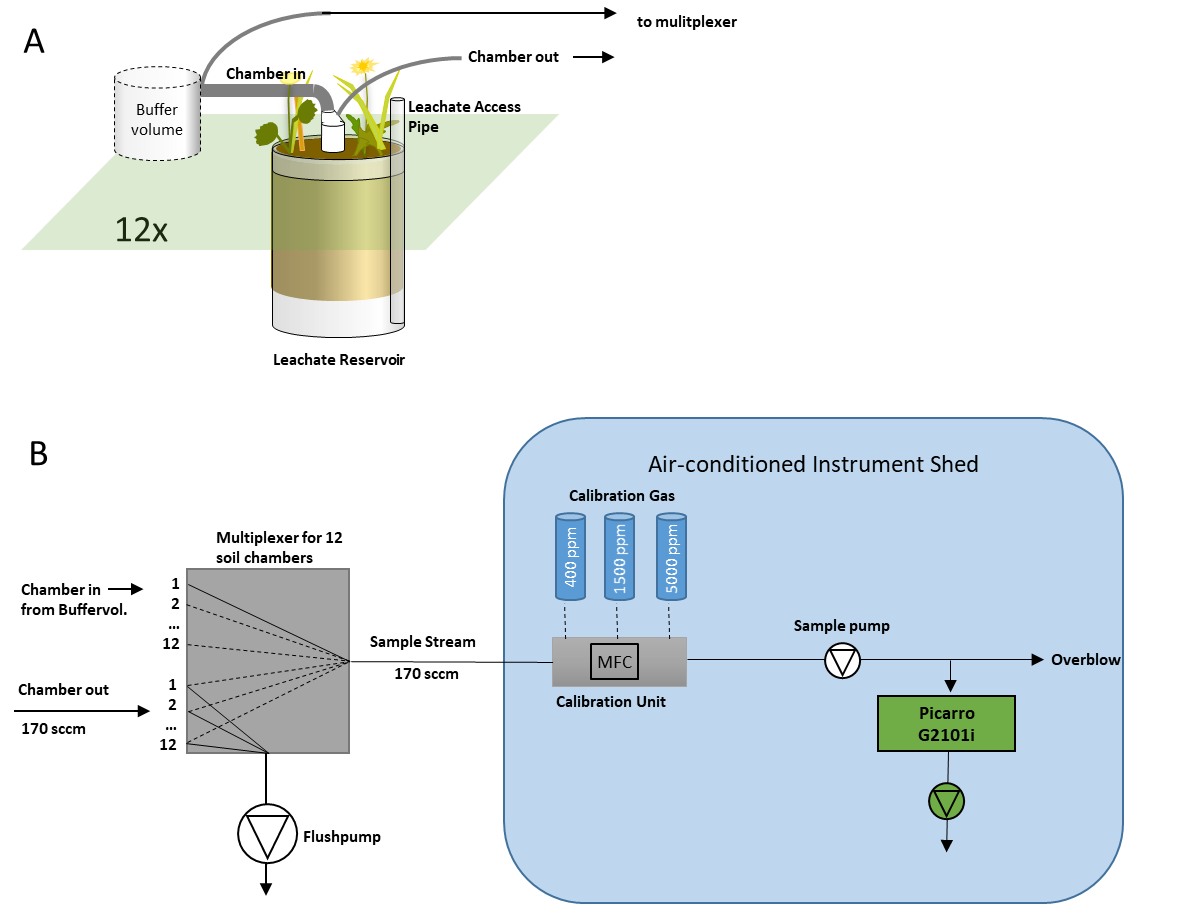


**Fig S2: ^13^CO_2_ back-diffusion from soil after dark labeling.** ^13^CO_2_­ soil efflux, expressed as atom-fraction of ^13^C in total CO_2_ efflux from soil, immediately after dark labeling of monoliths of the (a) managed and (b) the abandoned grassland. Error bars denote SEM within 30s intervals. Black lines denote the exponential fit.


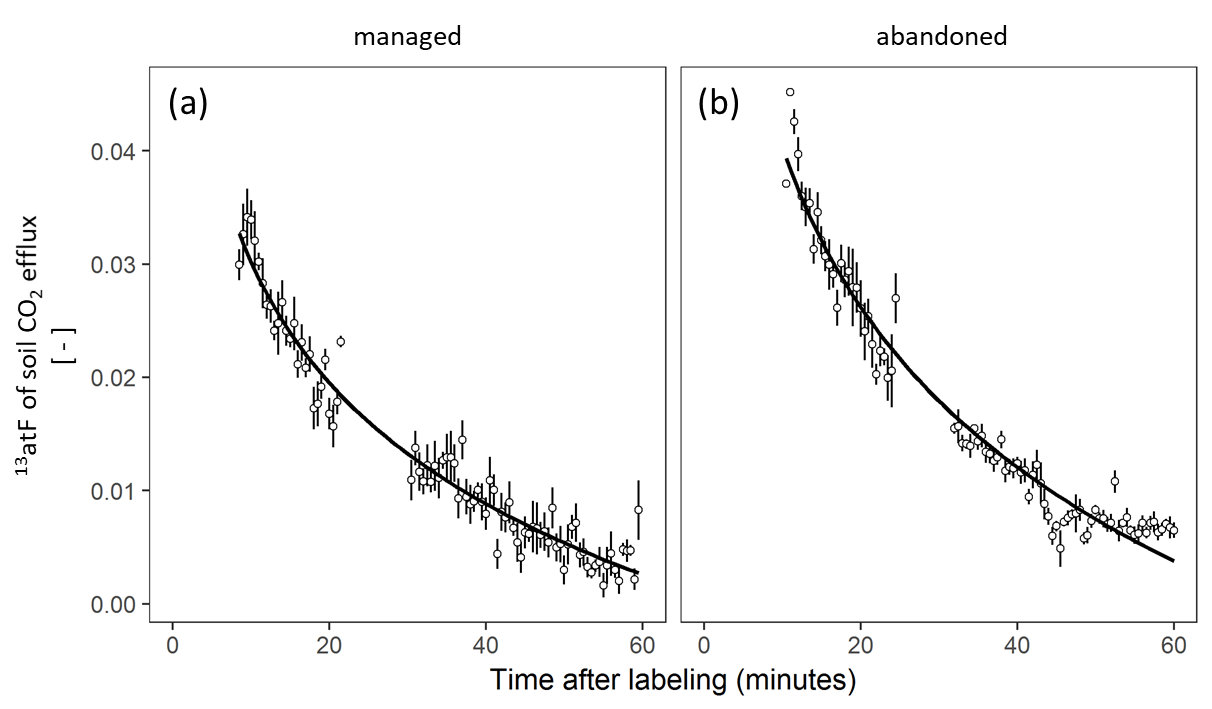


**Fig. S4** (a, b) Content and (c – f) incorporated ^13^C in (a, c, e) root sucrose and (b, d, f) root starch and fructan at peak drought (pD, 120 hours after labeling) and 3 days after the rewetting (+ 72h) in the control (blue) and drought (orange) treatment. Circles refer to the managed grassland, squares to the abandoned grassland. Error bars indicate ±SD. The shaded background indicates the drought treatment. Stars indicate significance of drought effect within land use (** p < 0.01, permutational one-way ANOVA). Data from (Karlowsky et al., 2018b), see Supplementary Information S1 for details.

**
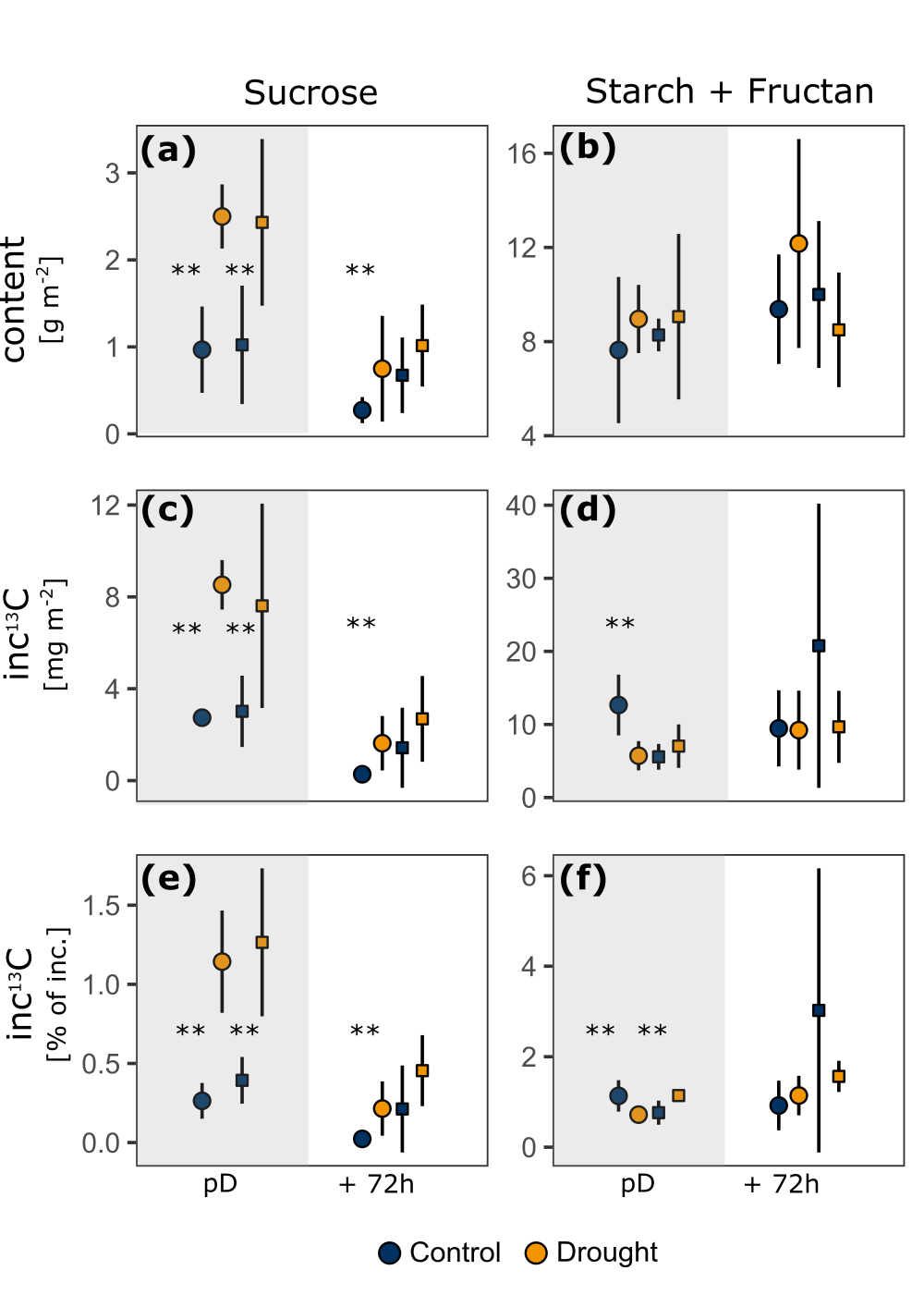
**

**Table S1** Model parameters of the exponential decay fit back-diffusion of atom-fraction excess of soil CO_2_ efflux during 60 minutes following the dark labeling. MRT = mean residence time

|  | Decay rate (±std. error)  min^-1^ | MRT  min |
| --- | --- | --- |
| meadow | - 0.04686 ± 0.00173 | 21.0 |
| abandoned grassland | - 0.03959 ± 0.00093 | 25.0 |

**Table S2** Effects of drought, land use and the interaction on the amount of root carbohydrates and incorporated ^13^C 120 hours after the peak-drought and the recovery labeling. Data from Karlowsky *et al*. 2018b (see Supplementary Information S1).

| **Campaign** | **Pool** |  | **content** | | |  | **inc. 13C (absolute)** | | |  | **inc. 13C (relative)** | | |
| --- | --- | --- | --- | --- | --- | --- | --- | --- | --- | --- | --- | --- | --- |
|  |  |  | F^a^ | p_F_^a^ | p_exact_^b^ |  | F^a^ | p_F_^a^ | p_exact_^b^ |  | F^a^ | p_F_^a^ | p_exact_^b^ |
| peak  Drought | Root sucrose | Drought | 14.705 | 0.005 | **<0.001** |  | 13.749 | 0.006 | **<0.001** |  | 25.730 | 0.001 | **<0.001** |
|  |  | Land use | 0.000 | 0.988 | 0.985 |  | 0.052 | 0.826 | 0.796 |  | 0.530 | 0.487 | 0.430 |
|  |  | Drought:Land use | 0.026 | 0.876 | 0.840 |  | 0.182 | 0.681 | 0.608 |  | 0.000 | 0.983 | 0.966 |
|  | Root  starch + fructan | Drought | 0.539 | 0.484 | 0.297 |  | 2.706 | 0.139 | *0.053* |  | 0.018 | 0.898 | 0.872 |
|  |  | Land use | 0.067 | 0.803 | 0.709 |  | 3.013 | 0.121 | **0.039** |  | 0.038 | 0.850 | 0.815 |
|  |  | Drought:Land use | 0.036 | 0.855 | 0.781 |  | 6.378 | 0.036 | **0.002** |  | 8.988 | 0.017 | **0.002** |
| Recovery | Root sucrose | Drought | 0.045 | 0.837 | 0.802 |  | 0.941 | 0.360 | 0.204 |  | 2.970 | 0.123 | *0.054* |
|  |  | Land use | 3.877 | 0.085 | **0.023** |  | 1.688 | 0.230 | 0.086 |  | 4.359 | 0.070 | 0.021 |
|  |  | Drought:Land use | 5.451 | 0.048 | **0.011** |  | 3.047 | 0.119 | **0.043** |  | 6.445 | 0.035 | **0.005** |
|  | Root  starch + fructan | Drought | 0.625 | 0.452 | 0.342 |  | 0.800 | 0.397 | 0.209 |  | 0.542 | 0.483 | 0.093 |
|  |  | Land use | 9.463 | 0.015 | **0.001** |  | 0.040 | 0.846 | 0.759 |  | 1.495 | 0.256 | 0.282 |
|  |  | Drought:Land use | 2.668 | 0.141 | *0.066* |  | 0.515 | 0.494 | 0.305 |  | 0.028 | 0.871 | 0.829 |

^a^F-values and approximate p-values from ordinary ANOVA; and ^b^ exact p-values from the permutational ANOVA (function ‘aovp’, R package ‘lmPerm’), bold values p_exact_ < 0.05.

**Table S3** Effects of drought and rewetting (time) on amount of root carbohydrates and incorporated ^13^C. Data from Karlowsky *et al*. 2018b (see Supplementary Information S1).

|  | **Fixed effect** |  | **content** | | |  | **inc. 13C (absolute)** | | |  | **inc13C (relative)** | | |
| --- | --- | --- | --- | --- | --- | --- | --- | --- | --- | --- | --- | --- | --- |
|  |  |  | DenDF | F | p |  | DenDF | F | p |  | DenDF | F | p |
| **Root Sucrose** | **Drought** |  | 18 | 17.965 | **<0.001** |  | 18 | 17.142 | **0.001** |  | 18 | 31.011 | **<0.001** |
|  | **Landuse** |  | 18 | 0.546 | 0.470 |  | 18 | 0.252 | 0.622 |  | 18 | 3.002 | 0.100 |
|  | **Time** |  | 18 | 22.573 | **<0.001** |  | 18 | 25.583 | **<0.001** |  | 18 | 30.307 | **<0.001** |
|  | **Drought:Landuse** |  | 18 | 0.085 | 0.774 |  | 18 | 0.168 | 0.687 |  | 18 | 0.012 | 0.914 |
|  | **Drought:Time** |  | 18 | 5.734 | **0.028** |  | 18 | 6.127 | **0.023** |  | 18 | 11.286 | **0.003** |
| **Root  Starch + Fructan** | **Drought** |  | 8 | 0.327 | 0.5833 |  | 18 | 1.541 | 0.2304 |  | 18 | 0.421 | 0.5245 |
|  | **Landuse** |  | 8 | 0.151 | 0.7078 |  | 18 | 0.195 | 0.6639 |  | 18 | 1.728 | 0.2052 |
|  | **Time** |  | 10 | 3.898 | 0.0766 |  | 18 | 1.796 | 0.1969 |  | 18 | 2.177 | 0.1573 |
|  | **Drought:Landuse** |  | 8 | 0.665 | 0.4384 |  | 18 | 0.033 | 0.8584 |  | 18 | 0.204 | 0.6568 |
|  | **Drought:Time** |  | 10 | 0.069 | 0.798 |  | 18 | 0.187 | 0.6703 |  | 18 | 0.376 | 0.5473 |

Results from mixed-model ANOVA. Satterthwaite approximation is given for denominator degrees of freedom (DenDF). Bold-values p < 0.05.

**References**

Bates, D., Mächler, M., Bolker, B., Walker, S. (2015). Fitting Linear Mixed-Effects Models Using lme4. *Journal of Statistical Software*, *67*(1), 1–48. https://doi.org/10.18637/jss.v067.i01

Karlowsky, S., Augusti, A., Ingrisch, J., Hasibeder, R., Lange, M., Lavorel, S., Bahn, M., Gleixner, G. (2018a). Land use in mountain grasslands alters drought response and recovery of carbon allocation and plant-microbial interactions. *Journal of Ecology*, *106*, 1230–1243. https://doi.org/10.1111/1365-2745.12910

Karlowsky, S., Augusti, A., Ingrisch, J., Hasibeder, R., Lange, M., Lavorel, S., Bahn, M.& Gleixner, G. (2018b). *Data from: Land use in mountain grasslands alters drought response and recovery of carbon allocation and plant-microbial interactions.* https://doi.org/10.5061/DRYAD.3S57P.3

Kuznetsova, A., Brockhoff, P. B., Christensen, R. H. B. (2017). lmerTest Package: Tests in Linear Mixed Effects Models. *Journal of Statistical Software*, *82*(13), 1–26. https://doi.org/10.18637/jss.v082.i13

R Core Team. (2018). *R: A Language and Environment for Statistical Computing*. Vienna, Austria. Retrieved from https://www.R-project.org/

Wheeler, B.& Torchiano, M. (2016). *lmPerm: Permutation Tests for Linear Models*. Retrieved from https://CRAN.R-project.org/package=lmPerm
